# Economic efficiency versus accessibility: Planning of the hospital landscape in rural regions using a linear model on the example of paediatric and obstetric wards in the northeast of Germany

**DOI:** 10.1186/s12913-019-4016-2

**Published:** 2019-04-24

**Authors:** Neeltje van den Berg, Franziska Radicke, Ulrike Stentzel, Wolfgang Hoffmann, Steffen Flessa

**Affiliations:** 1 University Medicine Greifswald, Institute for Community Medicine, Ellernholzstrasse 1-2, 17489 Greifswald, Germany; 2 University of Greifswald, Chair of General Business Administration and Health Care Management, Friedrich-Loeffler-Strasse 70, 17487 Greifswald, Germany

**Keywords:** Regional hospital planning, Economic efficiency, Care close to residence, Linear model, paediatric wards, obstetric wards

## Abstract

**Background:**

Costs for the provision of regional hospital care depend, among other things, on the population density and the maximum reasonable distance to the nearest hospital. In regions with a low population density, it is a challenge to plan the number and location of hospitals with respect both to economic efficiency and to the availability of hospital care close to residential areas.

We examined whether the hospital landscape in rural regions can be planned on the basis of a regional economic model using the example which number of paediatric and obstetric wards in a region in the Northeast of Germany is economically efficient and what would be the consequences for the accessibility when one or more of the three current locations would be closed.

**Methods:**

A model of linear programming was developed to estimate the costs and revenues under different scenarios with up to three hospitals with both a paediatric and an obstetric ward in the investigation region. To calculate accessibility of the wards, geographic analyses were conducted.

**Results:**

With three hospitals in the study region, there is a financial gap of €3.6 million. To get a positive contribution margin for all three hospitals, more cases have to be treated than the region can deliver. Closing hospitals in the parts of the region with the smallest population density would lead to reduced accessibility for about 8% of the population under risk.

**Conclusions:**

Quantitative modelling of the costs of regional hospital care provides a basis for planning. A qualitative discussion to the locations of the remaining departments and the implementation of alternative healthcare concepts should follow.

## Background

In Germany, the provision of adequate inpatient healthcare is generally determined by federal law (§ 1 of the Hospital Financing Act) whereas hospital planning (including geographic location, number of beds, departments, wards, and specializations of the hospitals) is the responsibility of the 16 federal states of Germany. Hospital plans serve as an instrument to reach an adequate level of healthcare of inpatient care in determined regions [1].

In the catchment area of many rural hospitals, the population is decreasing and aging simultaneously. In consequence, hospitals in such regions have to face decreasing capacity utilization especially in paediatric and obstetric wards, which can cause both economic problems and a lower quality of medical care [2].

This situation may lead to a conflict between the necessity to concentrate capacities for quality and economic reasons and the need to provide for sufficiently available healthcare in rural regions. To find a transparent and fair balance between quality of care, cost effectiveness and accessibility, evidence-based planning of the hospital landscape is necessary.

### Theoretical background

Important parameters for hospital planning are the number of inhabitants and the population density in a region, as well as the age distribution of the regional population. The relationship between population density and number of beds has been the foundation of hospital planning as early as 1946 when the United States Congress passed the “Hospital Survey and Construction Act” which became well known as the Hill–Burton Act. The formula, which was used to determine the number of beds for a hospital or region, has currently become the standard of regional hospital planning and is the foundation of hospital plans in Germany. It calculates the demand for hospital beds of a specific department i in a given region [3]:

$$ {B}_i=\frac{P\bullet {h}_i\bullet {V}_i}{a_i\bullet 1000\bullet 365} $$

with

*B*_*i*_ *=* Number of beds of department i required

*P =* Population

*h*_*i*_ *=* Hospital admission rate of department i [per 1000 inhabitants]

*V*_*i*_ *=* Average length of stay in department i in days

*a*_*i*_ *=* Occupancy rate in department i

**Fig. 1:**
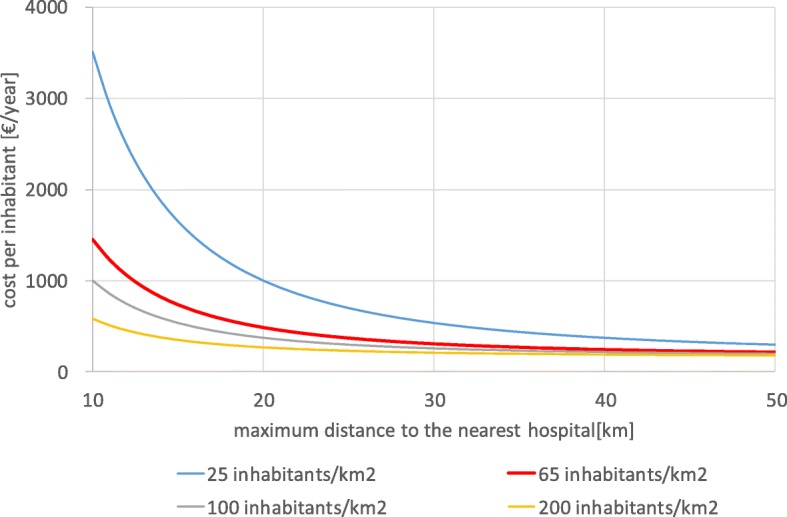
Hospital costs per inhabitant depending on maximum distance to the nearest hospital [4]

However, this formula ignores the close relationship between the distance to a hospital and treatment cost per inhabitant. Until today, no maximum distances from the residences of the patients to the hospitals have been officially defined in Germany, so that the number and locations of hospitals are more or less based on the inherited hospital system irrespective whether the location of hospitals is efficient. As hospitals have high fixed costs, efficiency would call for few big hospitals, while accessibility calls for many small hospitals spread throughout the country. The following formula calculates the hospital cost per capita in a region assuming hexagon-like catchment areas. The hospital cost per capita is a function of the maximum distance (r) which a patient would have to travel. Fig. 1 expresses this formula graphically, i.e., the annual hospital cost per inhabitant depending on reasonable maximum distances for different population densities. Lower population density and smaller distance to the nearest hospital cause higher yearly hospital costs per inhabitant [4]:

$$ \frac{K}{P}=\frac{AT}{P\bullet 3{r}^2\sin (60)}\bullet F+\mathrm{h}\bullet v $$

with

r *=* Maximum distance [km]

AT *=* Total area of the region [km^2^]

P *=* Population in the region

h *=* Admission rate

K *=* Total hospital cost [€]

F *=* Fixed cost per hospital [€]

v *=* variable cost per case [€]

### Research question

In this analysis, we consider different scenarios in a databased planning model of paediatric and obstetric wards in the rural region Western Pomerania in the Northeast of Germany. In 2016, this region had three hospitals with wards for paediatrics and obstetrics. One of the hospitals is a university hospital (paediatric and obstetrics wards each have 24 beds); the other two hospitals are small hospitals of primary care (paediatric wards: 18 and 16 beds, respectively; obstetrics wards: 11 and 6 beds, respectively). A long-running political discussion concerns the needed number and locations of the wards.

The main research question of this analysis is which number of paediatric and an obstetric ward in this region is economically efficient and what would be the consequences for the accessibility of paediatric and obstetric inpatient care when one or more locations would be closed. To examine this question, three different scenarios with up to three hospital wards of the respective medical discipline were analysed.

## Methods

To answer the research question, health-economic and geographical methods were combined. To address the economic parts of the research question, a model of linear programming (LP) was calculated to estimate the costs and revenues under different scenarios with up to three hospitals with both a paediatric and an obstetric ward in the investigation region [5, 6].

To calculate catchment areas and accessibility of the wards for the regional population, geographic analyses were conducted based on a geographical information system.

### Concept of efficiency

The model described in the following section maximizes the efficiency of a set of hospitals in a region. Our analysis takes the perspective of the provider (i.e. hospitals). Other perspectives, such as the society, financer (i.e. health insurance schemes) or patients are not considered in the model. Consequently, the model presented here cannot contemplate all aspects of economic efficiency and will in particular ignore some costs, such as transport costs for patients. In the sub-section “geographical analyses and population data” we will reflected on the distances which are partly reflecting the travel costs.

Generally, efficiency (*E*) can be defined as quotient of results (*R*) and resources; this quotient has to be maximized. If the results are defined as constant and the value of resources is expressed in costs (*C*), the efficiency quotient can be reduced to a cost minimization [7]. Our model assumes that the service units of the hospital(s) are given, i.e., all patients will be treated but the location of treatment might change, i.e.,

$$ E=\frac{Results}{Resources}\to \mathit{\operatorname{Max}}!\leftrightarrow \left\{\begin{array}{ll}C\to \mathit{\operatorname{Min}}!& if\ results= const.\\ {}R\to \mathit{\operatorname{Max}}!& if\ resources= const.\\ {}\frac{Results}{C}\to \mathit{\operatorname{Max}}!& else\end{array}\right. $$

The provider perspective allows reducing the efficiency to a return-on-investment (RoI) formula. The numerator are the revenues (price p times quantity q) of the hospital, the denominator are the fixed (*K*_*f*_*)* and variable costs (variable unit cost v times quantity q). Efficiency is maximized by maximizing this quotient. However, as both numerator and denominator are currency units, we can also reduce this problem to the maximization of the difference between revenues and costs. Under the assumption that a hospital does not challenge its complete existence but merely optimizes its service portfolio, fixed costs are not decision-relevant and the difference expresses the marginal contribution (*m*) defined as the difference between price and variable costs [8].

$$ E=\frac{Results}{Resources}= RoI=\frac{\sum {p}_j\bullet {q}_j}{K_f+\sum {v}_j\bullet {q}_j}\to \mathit{\max}!\leftrightarrow $$

$$ \pi =\sum {p}_j\bullet {q}_j-{K}_f-\sum {v}_i\bullet {q}_i\to \mathit{\max}!\leftrightarrow $$

$$ M=\sum {p}_j\bullet {q}_j-\sum {v}_j\bullet {q}_j=\sum \left({p}_j-{v}_j\right)\bullet {q}_j=\sum {m}_j\bullet {q}_j\to \mathit{\max}! $$

Consequently, we can reduce the problem of maximizing efficiency in a hospital system to a linear program under the assumption that we concentrate on the provider perspective and safeguard that results are constant [9]. At the same time, we have to assume that factor costs and variable costs are constant, i.e., models of production planning disregard economies or diseconomies of scale. Most models of production planning, furthermore, assume that fixed costs are given, but this is – as we will see in the following sub-section – no prerequisite.

### Linear program

About 50 years ago, a first model of linear programming (LP) was developed to allocate resources in hospitals efficiently. This development of a rational allocation model was almost in parallel with the development of the DRG (Diagnosis Related Groups) system by Robert B. Fetter at the Department of Operations Research of Yale University. DRGs describe a classification system for a lump sum billing procedure, with which hospital cases are assigned to case groups on the basis of medical data [10].

Further models followed [11–13].These models were scientifically interesting but of very little practical use as the computer capacity of that time prevented realistic LPs to be developed. In particular, the number of binary variables was limited. Consequently, several decades passed before the idea of calculating an optimum allocation in a hospital was taken-up again by Meyer in 1996 [14]. Later, Meyer & Harfner showed the potential of these models for horizontal integration but they never applied it to a regional system in rural areas. The model was more relevant for urban areas where accessibility was of no interest [6].

In principle, the model maximizes for each hospital the marginal contribution. The German hospital financing system is dual, i.e., buildings, vehicles and equipment are paid by the government while running expenditures are paid by the health insurance funds based on the G-DRG-system (German Diagnosis Related Groups). Thus, only running expenditure are relevant for this analysis. Depreciation for buildings, vehicles and equipment can be ignored. However, even among the costs recovered by the DRG, the majority is fixed or step-fixed, i.e., they remain stable for some variation of outputs and then jump to a higher level where they will be stable again until another threshold of outputs is reached so that they jump again. Our model distinguishes them accordingly (e.g. each department has fixed costs while personnel are step-fixed). The DRG itself is a price per service unit and does not distinguish fixed and variable costs [15, 16].

Each hospital can treat patients of n different DRGs and receives the respective rebate d_j_ (j = 1..n).

The direct cost per patient of DRG j (e.g. for food, drugs, implants etc.) are denoted by a_j_. Furthermore, fixed cost of FD_j_ have to be considered if at least one patient of DRG j is admitted and treated. Respective fixed costs could be salaries of midwives if the hospital offers vaginal deliveries. Several DRGs are combined to one department with department-specific fixed costs of FA_p_. All DRGs are allocated to the respective departments. Finally, the hospital has hospital fixed costs of FK, e.g. for administration. Table 1 gives an overview of a marginal contribution calculation of one hospital. x_j_ denotes the number of cases of DRG j. The simplified example assumes that DRGs 1 and 2 are allocated to department 1 whereas DRGs n, n-1 and n-2 are allocated to department b.

**Table 1:** Modell of marginal contribution analysis in a hospital

		DRG 1	DRG 2	DRG 3	DRG..	DRG n-2	DRG n-1	DRG n
Revenues		x_1_**⋅**d_1_	x_2_**⋅**d_2_	x_3_**⋅**d_3_	…	x_n-2_**⋅**d_n-2_	x_n-1_**⋅**d_n-1_	x_n_**⋅**d_n_
–	Direct Cost	x_1_**⋅**a_1_	x_2_**⋅**a_2_	x_3_**⋅**a_3_	…	x_n-2_**⋅**a_n-2_	x_n-1_**⋅**a_n-1_	x_n_**⋅**a_n_
=	Contribution I	x_1_**⋅**(d_1_-a_1_)	x_2_**⋅**(d_2_-a_2_)	x_3_**⋅**(d_3_-a_3_)	…	x_n-2_**⋅**(d_n-2_-a_n-2_)	x_n-1_**⋅**(d_n-1_-a_n-1_)	x_n_**⋅**(d_n_-a_n_)
–	DRG-fixed cost	FD_1_	FD_2_	FD_3_	…	FD_n-2_	FD_n-1_	FD_n_
=	Contribution II	x_1_**⋅**(d_1_-a_1_)- FD_1_	x_2_**⋅**(d_2_-a_2_)- FD_2_	x_3_**⋅**(d_3_-a_3_)- FD_3_	…	x_n-2_**⋅**(d_n-2_-a_n-2_)- FD_n-2_	x_n-1_**⋅**(d_n-1_-a_n-1_)- FD_n-1_	x_n_**⋅**(d_n_-a_n_)- FD_n_
–	department cost	FA_1_		…		FA_b_		
=	Contribution III	x_1_**⋅**(d_1_-a_1_)- FD_1_ + x_2_**⋅**(d_2_-a_2_)- FD_2_ - FA_1_		…		x_n-2_**⋅**(d_n-2_-a_n-2_)-FD_n-2_ + x_n-1_**⋅**(d_n-1_-a_n-1_)- FD_n-1_ + x_n_**⋅**(d_n_-a_n_)-FD_n_ – Fa_b_		
–	hospital-fixed cost	FK						
=	profit/loss	$\sum \limits_{j=1}^n\left({d}_j-{a}_j\right)\cdot {x}_j-\sum \limits_{j=1}^n{FB}_j-\sum \limits_{p=1}^b{FA}_p- FK$						

The model used for this paper assumes that the objective function given in the last row of Table 1 is maximized by each hospital while assuming that that total demand of patients in the area is met. If we maximize the objective function for each institution independently, it is likely that hospital specialize in a way that some DRGs are not covered [17, 18]. However, if all hospitals in a region behave in this pattern the needs of the population will not be met. Consequently, the model assumes that a region is covered by s hospitals in cooperation and that all cases must be treated. At the same time, we assume that the same DRGs are allocated to the same departments in all hospitals. The model defines the following variables:

*x*_*jk*_ *=* Number of treated patients in DRG j in hospital k, j = 1..n; k = 1..s; integer

*K*_*ik*_ *=* Units of resource i in hospital k, i = 1..m; k = 1..s

*ß*_*jk*_ $$ =\left\{\begin{array}{cc}1& if\  DRG\ j\  is\ in\ the\ service\ portfolio\ of\ hospital\ k\\ {}0& else\end{array}\right. $$, j = 1..n; k = 1..s

*D*_*pk*_ $$ =\left\{\begin{array}{cc}1& if\ department\ p\  is\ opened\ in\ hospital\ k\\ {}0& else\end{array}\right. $$, *p* = 1..b; k = 1..s

*DTotal*_*k*_ $$ =\left\{\begin{array}{cc}1& if\ hospital\ k\  is\ opened\\ {}0& else\end{array}\right. $$, k = 1..s

and constants:

*k*_*ik*_ *=* Capacity per service unit of resource i in hospital k, i = 1..m; k = 1..s

*c*_*ijk*_ *=* Consumptoin of resource i for one unit of DRG j in hospital k, j = 1..n; i = 1..m; k = 1..s

*d*_*j*_ *=* Rebate of DRG j, j = 1..n

*a*_*jk*_ *=* direct cost of one case of DRG j in hospital k, j = 1..n; k = 1..s

*nv*Number of DRGs

*M =* $$ M\in N, with\ M>\sum \limits_{j=1}^n\sum \limits_{k=1}^s{x}_{jk} $$

*b =* Number of departments

*R*_*p*_ *=* set of DRGs treated in department p, p = 1..b

*FD*_*jk*_ *=* DRG-specific fixed cost in hospital k, j = 1..n; k = 1..s

*FA*_*pk*_ *=* department-specific fixed cost of p in hospital k, p = 1..b; k = 1..s

*FK*_*k*_ *=* hospital fixed cost in hospital k; k = 1..s

*B*_*j*_ *=* number of patients in DRG j, j = 1..n

*w*_*ik*_ *=* Cost per unit of resource i in hospital k, i = 1..m; k = 1..s

The LP maximizes the total marginal contribution in the entire region:

$$ \sum \limits_{k=1}^s\sum \limits_{j=1}^n\left({d}_j-{a}_{jk}\right)\bullet {x}_{jk}-\sum \limits_{k=1}^s\sum \limits_{j=1}^n{FD}_{jk}\bullet {\beta}_{jk}-\sum \limits_{k=1}^s\sum \limits_{p=1}^b{FA}_{pk}\bullet {D}_{pk} $$

$$ -\sum \limits_{k=1}^s{FK}_k\bullet {DTotal}_k-\sum \limits_{k=1}^s\sum \limits_{i=1}^m{w}_{ik}\bullet {K}_{ik}\to \mathit{\operatorname{Max}}! $$

subject to the constraints:

$$ I.\kern0.5em \sum \limits_{j=1}^n{c}_{ijk}\bullet {x}_{jk}\le {k}_{ik}\bullet {K}_{ik}, for\ i=1..m,k=1..s $$

$$ II.\kern0.5em {x}_{jk}\le M\bullet {\beta}_{jk}, for\ j=1..n,k=1..s $$

$$ III.\sum \limits_{j\in {R}_p}{x}_{jk}\le M\bullet {D}_{pk}, for\ p=1..b,k=1..s $$

$$ IV.\sum \limits_{j=1}^n{x}_{jk}\le M\bullet {DTotal}_k, for\ k=1..s $$

$$ V.\sum \limits_{k=1}^s{x}_{jk}={B}_j, for\ j=1..n $$

The first constraints safeguards that the capacity limitations are respected in each hospital. The second determines that the binary variable ß_jk_ is one if at least one patient is treated with DRG j in hospital k so that the DRG-specific fixed costs are reflected in the objective function. The third and fourth constraints do the same to the department and hospital fixed costs. Finally, the last equation safeguards that all patients are treated in the region.

### Health economic data

Table 2 shows the parameters of the model based on the following assumptions:

1. Paediatric wards [16, 19, 20]:

- Nursing care has to be available 365 days a year, 24 h a day. During the core time (two shifts a day) two nurses, otherwise one. With a weekly working time of 40 h and 8 weeks of absence from work (holiday, training, illness) this results in a minimum of nine nurses.
- An average nursing care of 5.2 h per day and patient and an average hospital stay of 3.1 days was assumed (data from the hospital controlling department).
- One paediatrician specialist and one paediatrician in training have to be available at any time. This results in a minimum staffing of five physicians plus a senior physician.
- 45 min physicians’ time per day per patient for medical history, diagnostics, therapy decisions, monitoring, and documentation are assumed.

2. Obstetric wards [16, 19, 20]:

- Midwives have to be available 365 days a year, 24 h a day. With a weekly working time of 40 h and 8 weeks of absence from work (holiday, training, illness) this results in a minimum staffing of five midwifes. Since normally the head of the ward (senior midwife, included in the fixed costs) also cares for births, the minimum staffing can be reduced to four midwifes.
- The duration of a birth takes 14.5 h on average, considering all modes of delivery (spontaneous or assisted vaginal delivery, caesarean section).
- It is assumed that the number of delivery rooms is sufficiently large (no capacity limitation).
- As in the paediatric ward, a minimum staffing of nine nurses is necessary.
- For each patient, nursing care of 2.8 h per day plus 1.8 h per day for a new-born is assumed.
- The caesarean section rate is 35.6%. This result in an average length of stay of 4.9 days (normal birth: 3.6 days, caesarean section: 7.3 days).
- The minimum staffing is five doctors and one senior physician, analogue to the paediatric ward.
- The assumed physician-working time per vaginal delivery is 60 min.
- The assumed physician-working time for a caesarean section is 300 min (including 120 min anaesthesiologist). Additionally, 300 min nursing care are needed.

3. Salaries: For nurses and midwifes, a gross annual salary of 42,000€ was assumed, for senior nurses and midwifes (management of the ward) 50,000. Physician specialists in training cost 75,000€/year, senior physician specialists 120,000€. 35% employer’s share has to be added.
4. Department fixed costs: Fixed costs per department are added for administration, cleaning, heating etc. We calculate €50,000/year per ward and 35,000€/year per delivery room. Including the salaries (which are fixed as well) these assumptions result in fixed costs for the wards of 576,500€/year for paediatric wards and 679,000€/year for obstetric wards.
5. Fixed cost per bed: The second kind of costs is fixed costs per bed. Here, an exact calculation was not possible. Therefore, we used average values for paediatrics and obstetrics that are available in the context of the calculation of DRG-values for a normal birth, a caesarean section, a healthy newborn, and a new-born, born by a caesarean section. The fixed costs per bed are 15,253.47€/year for beds on the paediatrics wards and 20,212.24€/year for beds on the obstetrics wards.
6. Variable costs: The third kind of costs are variable costs (e.g. drugs, food). These include according to the average DRG-values

- Paediatrics: 152.59€/case
- Obstetrics: 244.64€/case

7. Revenues: The revenues for the cases are calculated based on the base case value of the Federal State of Mecklenburg-Western Pomerania (3117.36€) and the case mix indices for paediatrics (0.483) and births (1.006). This results in revenues of 1505.68€ per case in paediatrics and 3135.10€ per birth.

**Table 2:** Basic parameters of the model

Number of hospitals		*3*	
Number of DRGs		*2*	
	j = 1	*delivery*	
	j = 2	*paediatrics*	
Capacity of personal category i in hospital k, i = 1..5, k = 1..3		*k*_*ik*_	
	i = 1	1760 h	
	i = 2	1760 h	
	i = 3	1760 h	
	i = 4	1760 h	
	i = 5	1760 h	
Time consumption of personal category i for production of one unit of DRG j in hospital k, j = 1..2; i = 1..5; k = 1..3		j = 1	j = 2
	1	16.2	
	2	2.33	
	3		14.50
	4		22.62
	5		1.00
Rebate of DRG j, j = 1..2	d_1_ = 1505.68 d_2_ = 3135.10		
Direct cost for one case of DRG j in hostpial k, j = 1..2; k = 1..3	a_1,k_ = 152.59 a_2,k_ = 244.64		
Department fixed costs of department j in hospital k, j = 1..2; k = 1..3	*FA*_*1k*_ = 576,500 *FA*_*2k*_ = 679,000		
Fixed cost per bed for DRG j in hospital k, j = 1..2; k = 1..3	*bA*_*1k*_ = 15,253.47 *bA*_*2k*_ = 20,212.24		
Cost per staff of category i in hospital k, i = 1..5; k = 1..3		*w*_*ik*_	
	i = 1	56,700	
	i = 2	101,250	
	i = 3	56,700	
	i = 4	56,700	
	i = 5	101,250	
Average lengths of stay in DRG j, j = 1..2	*v*_*1*_ = 3.10 *v*_*2*_ = 4.92		

### Geographical analyses and population data

To identify the potential number of patients in the study region, we calculated catchment areas of the hospitals using a Geographic information System (ArcGIS 10.0 (ESRI, Redlands, USA)). To calculate the catchment areas, it was assumed that patients visit the paediatric or obstetric ward in the nearest hospital. The travel time to the nearest hospital was calculated using the centre points of the municipalities and municipal districts as origins of the patients. The travel time to the hospital was determined alongside the road network. We included other hospitals with paediatric and obstetric wards in neighbouring regions to get realistic catchment areas.

The number of cases in outpatient paediatrics and obstetrics in the postal code areas of the study region were retrieved from the InEK-database of the Federal State of Mecklenburg-Western Pomerania (InEK: Institut für das Entgeltsystem im Krankenhaus, in English: Institute for the hospital remuneration system). In this database, the numbers of all different DRGs are available on the level of postal code areas. These data give information on the number of cases in the population of a postal code region, not on the hospital where the DRGs were remunerated. The cases were assigned to the respective catchment areas of the hospitals using the Geographic information System. The numbers of cases in the catchment areas are the potential number of cases under the prerequisite that all patients consult the nearest hospital. The potential number of cases in the catchment area of the hospital is an indication for the limitations of the models: it is not realistic that the wards can acquire far more cases than the potential number of cases in the catchment area.

## Results

**Fig. 2:**
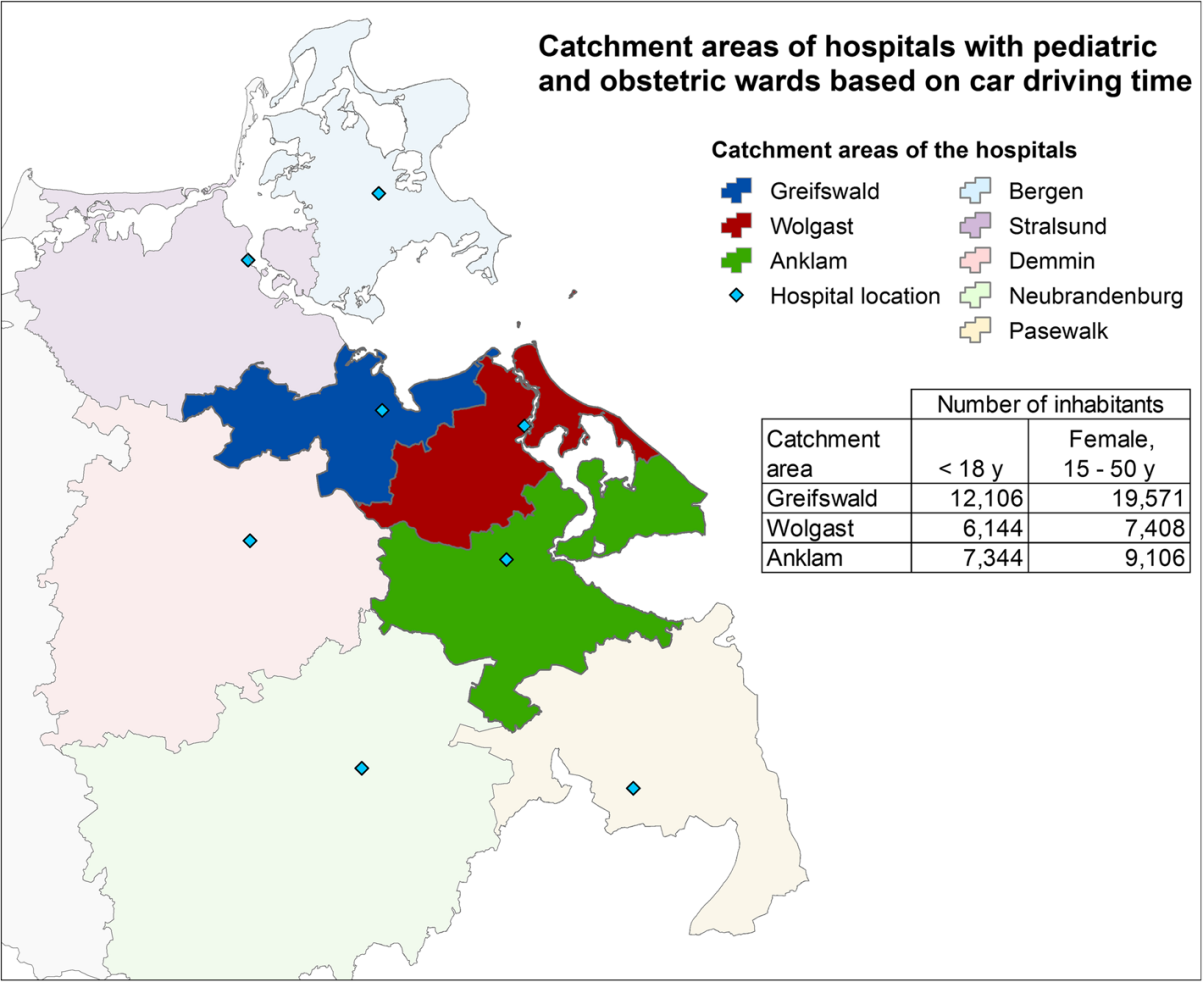
Catchment areas of the hospitals with paediatric and obstetric ward based on car driving time and associated number of inhabitants

Figure 2 shows the calculated catchment areas of the three hospitals in Greifswald, Wolgast, and Anklam.

To illustrate the current situation, Table 3 shows the number of cases in the catchments areas of the hospitals compared to the number of cases of the respective wards in the hospitals. The wards in the hospitals in Greifswald and Wolgast remunerated in 2014 more cases than available in the catchment area, in Anklam, fewer cases were remunerated.

**Table 3:** Case numbers of the hospitals and in in the catchment areas based on hospital data and on regional data; Data sources: Controlling departments of the hospitals, InEK (Institute for hospital remuneration) 2014

	Pediatrics		Obstetrics	
	Number of cases in the hospital	Cases in the catchment area	Number of births in the hospital	Births in the catchment area
Greifswald	1820	1192	800	518
Wolgast	1057	926	357	203
Anklam	496	898	280	293
Total	3373	3016	1437	1014

### Basic model including all three hospitals

Table 4 shows the results of the basic linear model including both wards in all three hospitals under realistic conditions. In this scenario, all wards work under their capacity and have a negative contribution margin, except for the paediatric ward in Greifswald. The overall deficit for the paediatric wards is € 1,6 million, for the obstetric wards € 2,8 million.

**Table 4:** Contribution margin including the wards in all three hospitals

	Pediatric wards				Obstetric wards				Total [€]
	Beds	Cases	Capacity utilization	Contribution margin [€]	Beds	Cases	Capacity utilization	Contribution margin [€]	
Wolgast	18	1057	50%	− 494,091	11	357	44%	−1,169,487	−1,663,578
Anklam	16	496	26%	−1,165,970	6	280	63%	−1,290,992	−2,456,962
Greifswald	24	1820	64%	49,899	24	800	45%	− 378,569	− 328,670
Total	58	3373	47%	−1,610,162	41	1437	51%	−2,839,048	−4,449,210

Table 5 shows the results of the basic model under the assumption that the number of beds is reduced to obtain 100% capacity utilization. However, also in this scenario there is an overall deficit of € 3,6 million for all three hospitals.

**Table 5:** Contribution margin including the wards in all three hospitals while optimizing the number of beds to obtain full capacity utilization

	Pediatric				Obstetric				Total [€]
	Beds	Cases	Capacity utilization	Contribution margin [€]	Beds	Cases	Capacity utilization	Contribution margin [€]	
Wolgast	9	1057	100%	− 356,810	5	357	100%	−1,048,214	−1,405,024
Anklam	5	496	100%	− 998,182	5	280	100%	−1,270,780	−2,268,962
Greifswald	16	1820	100%	171,927	11	800	100%	−115,809	56,118
Total	30	3373	100%	−1,186,065	21	1437	100%	−2,434,803	−3,617,868

### Scenarios with 1 and 2 hospitals

A linear model was calculated concentrating all paediatric cases and births in one hospital by adding the following constraints:

$$ \sum \limits_{k=1}^3{\beta}_{jk}=1, for\ j=1,2 $$

If all existing cases were treated in the same hospital under the condition of 100% capacity utilization, there would be a positive contribution margin. With full capacity utilization, this one hospital would generate a contribution margin of € 2.1 million instead of a deficit (Table 6).

**Table 6:** Cases form baseline at 2014 treated at one hospital, minimum number of beds

	Pediatric ward				Obstetric ward				Total [€]
	Beds (N)	Cases (N)	Capacity Utilization	Contribution Margin [€]	Beds (N)	Cases (N)	Capacity Utilization	Contribution Margin [€]	
Hospital	29	3373	100%	1,281,188	20	1437	100%	806,408	2,087,597

Since we had the assumption that the services offered in the three hospitals are equivalent, the location of the hospital does not matter if we only consider financial aspects. A concentration on two hospitals generates a negative result of − 237,000 € (data not shown).

### Accessibility of the hospitals

With three hospitals in the region, 15% of the children < 18 years and 14% of the women between 15 and 50 years have more than 20 min travel time to the nearest hospital. However, all patients reach the nearest hospital within 30 min travel time.

**Fig. 3:**
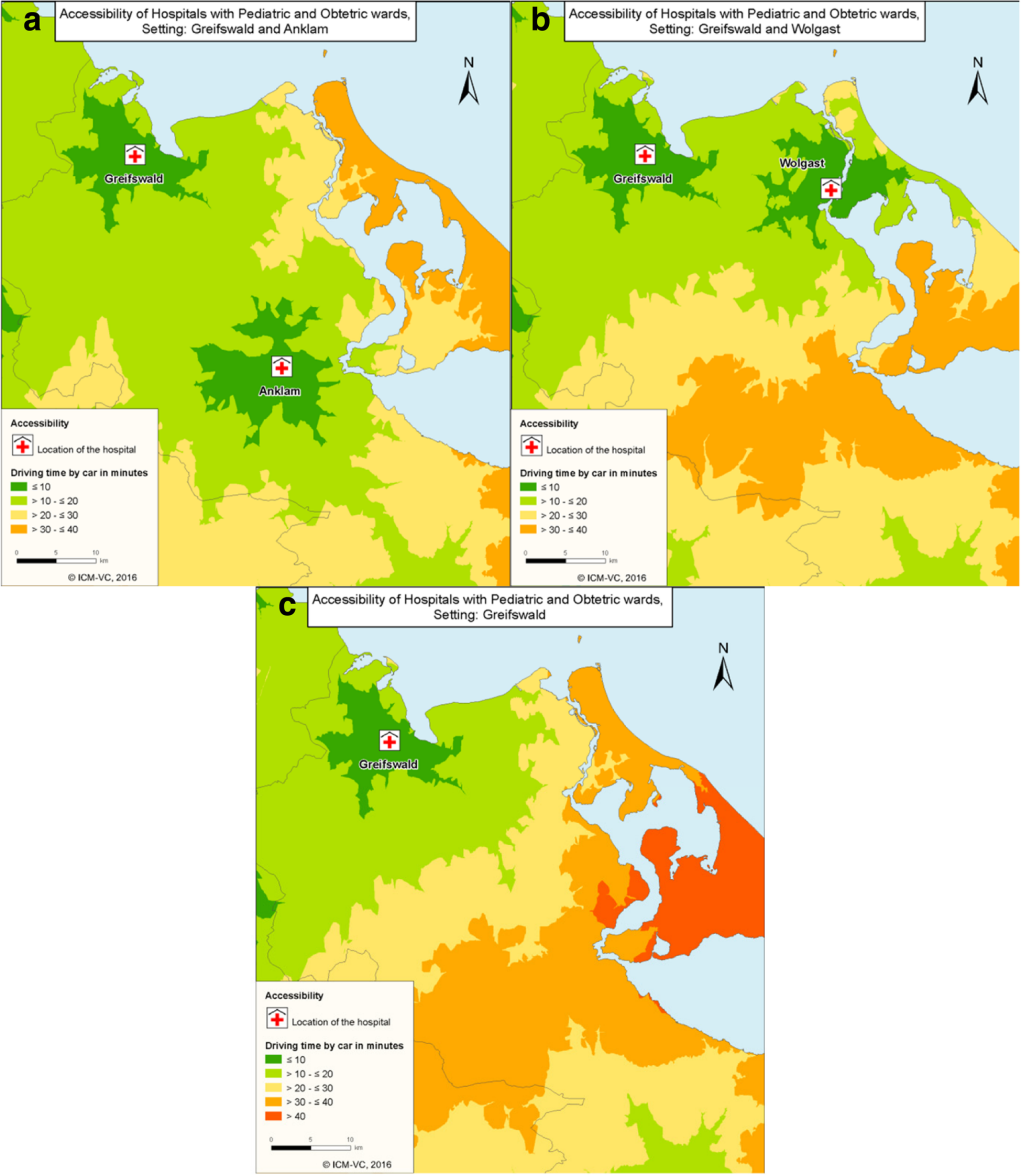
Accessibility by car to the hospitals with pediatric and obstetric wards in different settings: **a** Preserving the wards in Greifswald and Anklam; **b** Preserving the wards in Greifswald and Wolgast; **c** Concentration in one remaining hospital in Greifswald

Figure 3 illustrates the accessibility by car for three different scenarios according to the results of the linear programming model. Figure 3 (a) and (b) show the accessibility with one hospital location in Greifswald and one other hospital either in Anklam (A) or in Wolgast (B). The accessibility by car for a centralization of the cases in Greifswald under these models is shown in Fig. 3 (c).

If the obstetric and paediatric wards have their locations in Greifswald and Anklam (map a) or in Greifswald and Wolgast (map b) it is possible to reach the nearest hospital in at most 40 min for all patients in the region. If only the location in Greifswald would remain (Fig. 3 (c)) a part of the patients would have a longer drive by car. This concerns mainly people from the island Usedom, in the most eastern part of the study region. About 8% of the inhabitants under 18 years (*n* = 2060) of the study region and 8% of the women between 15 and 50 years (*n* = 2800) would be affected by travel times of more than 40 min by car.

## Discussion

The findings resulting from the LP model stress the conflict between accessibility (expressed in distances to the health care provider) and efficiency (expressed in cost per patient or inhabitant) which has been discussed frequently. For instance, Berwick et al. discuss the “triple” aim of health care, i.e. “improving the individual experience of care; improving the health of populations; and reducing the per capita costs of care for populations” while physical accessibility is a major factor of “improving the health of populations” [21]. This conflict was also a drive of the Declaration of Alma Ata on Primary Health Care in 1978 as an innovative way to reconcile this conflict inspiring the discussion on distributional ethics in health care [22–24]. Other authors stress that this conflict is the health-related expression of the general trade-off between equality and efficiency which is an underlying principle of economics [25–27]. However, many of these papers and discussions remain on a theoretical and in particular non-empirical level. The results presented in this paper demonstrate – based on the example of a remote German region – the conflict between costs of providing services and accessibility with real data.

The current discussions in Germany focus on the question whether the high number of small and unprofitable hospitals is still needed and should be subsidized by the government. The number of hospitals in Germany has been declining from 2411 hospitals in 1991 to 1956 in 2015 [19]. The closures mostly affect unspecialized hospitals in particular (but not only) in rural areas as they are seen as less efficient [28]. The linear models in this analysis showed a similar effect: too many hospitals with identical departments in a region with a low population density and, consequently, a low number of potential patients endanger the economic efficiency of the hospitals.

The basic model demonstrates a critical economic situation. Even if all three hospitals in the study region operate at the maximum capacity utilization, there is still a coverage gap of € 3.6 million. The optimization model points out that the case number is too small to allow positive financial results for more than one hospital. To get a positive contribution margin, every hospital would have to treat at least 894 births (13 beds) und 1587 paediatric cases (14 beds), which the region cannot deliver. Consequently, an analysis based only on the provider perspective would call for a consequent closure of the paediatric and obstetric departments in the two smaller hospitals.

However, the Government of the German states hesitate to base their decisions on mathematical models. For instance, Kuntz et al. developed a DEA-based model allowing efficiency-based resource allocation for one west-German state but the results were never applied [29, 30].

A consequence of closing down hospitals is that a part of the patients have a longer travel time to the next hospital, which could have an influence on access and utilization. In an analysis of reimbursement data of statutory health insurances, it was stated, that younger patients (< 30 years) on average travel longer distances than older patients. Patients in rural regions have twice as long travel times compared to patients in urban areas [31]. Stentzel at al examined in the same study region as our analysis (Western Pomerania), whether a longer travel time to an inpatient gynaecologist or GP practice leads to a lower utilization of those providers. However, no significant association was found here [32]. In a systematic review on minimum standards for spatial accessibility of primary care, different results on reasonable travel times from the USA, Germany and Austria were included. It was found that a travel time of 30 min for primary care for at least 90% of the population is acceptable from the perspective of the patients. The accepted travel time tends to be lower in urban regions [33]. Comparing these results to the geographic results of our analyses, the travel times to the nearest hospital might be ok for most of the population also in case of closing down one of the smaller hospitals with accordingly longer travel times and travel costs.

The results of the model presented here were considered by the government of the Federal State of Mecklenburg-Western Pomerania but were not highly influential. The attempt to close down the paediatric and obstetric departments in the two smaller hospitals inspired some “civil unrest” including demonstration walks and high election results for a right-wing partly. Consequently, the government had to consider much more aspects than only the costs and efficiency of hospitals.

As stated above, models of linear programming are frequently used for production planning. However, they have some limitations, which are also relevant for the interpretations of the results presented here. The following limitations should be considered carefully and call for further research:

- Perspective: The LP models takes the perspective of the provider and does not consider societal, financer or patient perspectives. Thus, it can only optimize the system from the perspective of the providers (and partly of the financers), while other costs (e.g. transport of patients) are not include in the model.
- Constant service units: the model assumes that the number of service units in the catchment area of the three hospitals is constant. In reality, the demand will also depend on the travel distance. For a model with only three hospitals and a catchment area where all hospitals are accessible in reasonable distances, this is acceptable. Extending the model to bigger regions and more hospitals would require the definition of a distance decay curve or a maximum travel distance.
- Linearity: Linear programming assumes that all functions are linear. Consequently, economies or diseconomies of scale cannot be considered. At the same time, the models consider efficiency gains only through the digression of the fixed costs. Efficiency gains through learning effects (e.g. more routine because of larger numbers of cases) could not be included.
- Decision-Model: The linear model optimizes the efficiency under certain constraints. However, it does not allow comparing the relative efficiency of hospitals based on empirical data. Other methods, such as Data Envelopment Analysis (DEA) and Stochastic Frontier Analysis (SFA) are designed to find the bench-marks. Consequently, DEA and SFA can give interesting insights into relative efficiency based on empirical data. They might be in particular helpful to compare the efficiency of the hospitals before and after the recommendations are implemented. This calls for further research.
- Quality assumption: The linear model assumes that the quality of services which can be provided in all three hospitals is equal and does not depend on volume. This is an assumption, but our experience with “normal deliveries” and “general pediatrics” underlines that this assumption is correct.
- Data: For the calculation of the models, average values for Germany were used to calculate costs because real data was only partly available. The salaries of nurses, midwifes, and physicians are based on collective agreements, these data are quite valid. Although the salaries between the hospitals might be comparable, there are differences in the structure of the staff between a university hospital and small regional hospitals. Other fixed and variable costs are likely to be different among the hospitals. Therefore, real comparability between the hospitals is limited.
- Catchment area: The assumption for the calculation of the catchment areas, that all patients visit the nearest hospital, is certainly not completely valid. Patients may be willing to travel longer distances to be treated in the university hospital or to give birth in a hospital with special offers.

Consequently, one should not over-estimate the relevance of one economic model. Other approaches [34, 35] and in particular a political analysis should be used in addition. Based on the limitations given above, other models could reflect on factors such as:

- Accessibility: which location has a good accessibility for the inhabitants of the region both by car and public transport;
- Availability of paediatric and obstetric wards in neighbouring regions;
- Medical equipment of the hospital where the paediatric and obstetric wards are located: a better medical equipment of the hospital could allow the treatment of more severe or complex patients;
- Other wards and departments in the hospital: it should be assessed, in which hospital the paediatric and obstetric wards fit best in the entire portfolio of health services of the hospital;
- Social and economic factors in the region.

Consequently, the trade-off between equity and efficiency must be solved by innovative forms of health care provision to ensure inpatient healthcare close to the homes of the patients despite the necessity of economic efficiency of the hospitals. Such innovative healthcare concepts for rural regions could comprise:

- A close cooperation between small hospitals in rural regions and a compensatory alignment of the services and wards;
- Conversion of hospitals into regional ambulatory healthcare centers to grossly reduce fixed costs [36, 37];
- A close cooperation between inpatient and outpatient providers with mutual support and compensation of services, For example is it possible to support the cooperation between outpatient midwifes and obstetric wards to ensure obstetric care in rural regions [38];
- Implementation of tele-medical connections between small hospitals and hospitals with maximum care to ensure medical standards in small hospitals maintaining only few medical specialties [39].
- Improvement of location in the public road system as well as public transport to and from hospitals [2].
- Improvement of emergency systems in order to safeguard rapid transport from the homes of patients to the hospital [40].

## Conclusion

Summarizing we can conclude that the conflict between accessibility and hospital cost per patient is obvious. Rural areas require a higher number of smaller hospitals in order to safeguard acceptable access times, but this will lead to costs and losses in hospitals challenging their existence. This conflict must be expressed and discussed in a transparent way. Mathematical modelling and geographic information systems are an excellent way to base these discussions on transparency and facts. However, even in a transparent process the conflict will not be solved unless innovative forms of health care delivery are developed and applied. This is a call for all health policy makers to invest creativity into regional hospital planning going beyond the hospital.

## Abbreviations

DEA: Data Envelopment Analysis
DRG: Diagnosis Related Groups
G-DRG: German Diagnosis Related Groups
InEK: Institut für das Entgeltsystem im Krankenhaus (in English: Institute for the hospital remuneration system)
LP: Linear programming
SFA: Stochastic Frontier Analysis

## References

[1] Krankenhausplan 2012 des Landes Mecklenburg-Vorpommern. 2018. Available at: https://www.regierung-mv.de/Landesregierung/wm/gesundheit/Gesundheitsversorgung/Krankenhauswesen/. Accessed 9 Jul 2018.

[2] Kozhimannil KB, Hung P, Prasad S, Casey M, McClellan M, Moscovice IS (2014). Birth volume and the quality of obstetric care in rural hospitals. J Rural Health.

[3] Trambacz J (2016). Grundlagen und Lehrbegriffe der Gesundheitsökonomie. Lehrbegriffe und Grundlagen der Gesundheitsökonomie.

[4] Fleßa S, Gieseler V, Herbst M, Dünkel F, Stahl B (2016). Die Rolle der Krankenhäuser im ländlichen Raum. Daseinsvorsorge und Gemeinwesen im ländlichen Raum.

[5] Fleßa S, Ehmke B, Herrmann R (2006). Optimierung des Leistungsprogramms eines Akutkrankenhauses : neue Herausforderungen durch ein fallpauschaliertes Vergütungssystem. BFuP.

[6] Meyer M, Harfner A (1999). Spezialisierung und Kooperation als Strukturoptionen für deutsche Krankenhäuser im Lichte computergestützter Modellrechnungen.

[7] Muennig P, Bounthavong M (2016). Cost-effectiveness analysis in health: a practical approach.

[8] Fleßa S (2018). Systemisches Krankenhausmanagement.

[9] Dantzig G (2016). Linear programming and extensions.

[10] Gurfield RM, Clayton SC (1969). Analytical hospital planning: a pilot study of resource allocation using mathematical programming in a cardiac unit.

[11] Shuman LJ, Young JP, Naddor E (1971). Manpower mix for health services – a prescriptive regional planning model. HSR.

[12] Shuman LJ, Wolfe H, Spears RD, Schniederjans MJ, Kwak N, Schmitz HH (1984). The role of operations research in regional health planning. Operations Research – Application in health Care planning:5.

[13] Dowling WL. Hospital production – a linear programming model. Toronto/London: Lexington; 1976.

[14] Meyer M (1996). Das optimale Fallklassen-Programm eines Krankenhauses. Führen und Wirtschaften im Krankenhaus.

[15] Vogl Matthias (2014). Hospital financing: Calculating inpatient capital costs in Germany with a comparative view on operating costs and the English costing scheme. Health Policy.

[16] Fallpauschalenkatalog. Available at https://www.g-drg.de/G-DRG-System_2018/Fallpauschalen-Katalog/Fallpauschalen-Katalog_2018. Accessed 22 Nov 2018.

[17] Ellis R, McGuire T (1996). Hospital response to prospective payment: horal Hazard, selection and practice style effects. J Health Econ.

[18] Newhouse JP (1996). Reimbursing health plans and health providers: selection vs. efficiency in production. J Econ Lit.

[19] Meinfeld H (2011). Personalbedarfsermittlung von Hebammen in Kliniken. Hebammenforum.

[20] DRG-Browser. Available at: http://www.g-drg.de/. Accessed 29 Mar 2018.

[21] Berwick DM, Nolan TW, Whittington J (2008). The triple aim: care, health, and cost. Health Aff.

[22] World Health Organization (WHO) (1978). Alma-Ata 1978: primary health care. Report on the International Conference on Primary Health Care, 6–12.

[23] Bankowski Z, Bryant JH, Gallagher J. Ethics, equity and health for all. Geneva: Publisher, CIOMS, WHO; 1997.

[24] Lindholm L, Rosen M, Emmelin M (1996). An epidemiological approach towards measuring the trade-off between equity and efficiency in health policy. Health Policy.

[25] Fleßa S. Gesundheitsreformen in Entwicklungsländern. Frankfurt am Main: Lembeck; 2003.

[26] Okun AM. Equality and efficiency - the big trade-off. Washington DC: The Brookings Institution; 1975.

[27] Berman PA (1982). Selective primary health care: is efficient sufficient?. Soc Sci Med.

[28] Fareed N (2012). Size matters: a meta-analysis on the impact of hospital size on patient mortality. Int J Evid Based Healthc.

[29] Staat M (2006). Efficiency of hospitals in Germany: a DEA-bootstrap approach. Appl Econ.

[30] Kuntz L, Scholtes S (1999). Wirtschaftlichkeitsanalyse mittels Data Envelopment Analysis zum Krankenhausbetriebsvergleich. Krankenhausmanagement.

[31] Schang L, Kopetsch T, Sundmacher L (2017). Travel times of patients to ambulatory care physicians in Germany. Bundesgesundheitsblatt Gesundheitsforschung Gesundheitsschutz.

[32] Stentzel U, Bahr J, Fredrich D, Piegsa J, Hoffmann W, van den Berg N (2018). Is there an association between spatial accessibility of outpatient care and utilization? Analysis of gynecological and general care. BMC Health Serv Res.

[33] Voigtländer S, Deiters T (2015). Minimum standards for the spatial accessibility of primary care: a systematic review. Gesundheitswesen..

[34] Jun GT, Morris Z, Eldabi T, Harper P, Naseer A, Patel B, Clarkson JP (2011). Development of modelling method selection tool for health services management: from problem structuring methods to modelling and simulation methods. BMC Health Serv Res.

[35] Pitt M, Monks T, Crowe S, Vasilakis C (2016). Systems modelling and simulation in health service design, delivery and decision making. BMJ Qual Saf.

[36] Landtag Mecklenburg-Vorpommern (2016). Älter werden in Mecklenburg-Vorpommern.

[37] Gesetz zur Reform der Strukturen der Krankenhausversorgung (Krankenhausstrukturgesetz – KHSG). 2015. Available at: https://www.bgbl.de/xaver/bgbl/start.xav?startbk=Bundesanzeiger_BGBl&jumpTo=bgbl115s2229.pdf#__bgbl__%2F%2F*%5B%40attr_id%3D%27bgbl115s2229.pdf%27%5D__1531164748012. Accessed at 9 Jul 2018.

[38] Hung P, Kozhimannil KB, Casey MM, Moscovice IS (2016). Why are obstetric units in rural hospitals closing their doors?. HSR.

[39] van den Berg N, Schmidt S, Stentzel U, Mühlan H, Hoffmann W (2015). The integration of telemedicine concepts in the regional care of rural areas: possibilities, limitations, perspectives. Bundesgesundheitsblatt Gesundheitsforschung Gesundheitsschutz.

[40] Fleßa S (2016). Der Telenotarzt als Innovation des Rettungswesens im ländlichen Raum–eine gesundheitsökonomische Analyse für den Kreis Vorpommern-Greifswald. Die Unternehmung.

